# Nonreciprocal transmission based on quasi-bound states in the continuum via scaled lattice constants

**DOI:** 10.1515/nanoph-2025-0320

**Published:** 2025-09-30

**Authors:** Ran Li, Junqiang Sun

**Affiliations:** Wuhan National Laboratory for Optoelectronics, Huazhong University of Science and Technology, Wuhan, China

**Keywords:** metasurface, Kerr nonlinearity, nonreciprocity intensity range, isolation, Fano resonance, Rabi splitting

## Abstract

Nonreciprocal devices are key technologies for modern photonic applications, and nonlinearly induced nonreciprocity based on metasurface platforms opens up the potential for compactness and miniaturization of such devices in free-space optical paths. In this work, a design of quasi-bound states in the continuum (QBICs) via scaled lattice constants is employed, featuring a plate-hole tetramer unit cell with single-step etching. Under normal incidence, QBICs exhibit polarization-insensitive excitation, with the scattering dominated by the combination term of magnetic dipoles, magnetic toroidal dipoles, electric quadrupoles as well as electric toroidal quadrupoles – rendering the vertical field distribution sensitive to parametric variations. Embedding Kerr nonlinearity yields nonreciprocal responses under upper and lower port excitation. The steep edges between peaks and valleys in the Fano-line-shaped transmission spectra facilitate balancing the nonreciprocal intensity range (NRIR) and isolation. The NRIR originates from electromagnetic asymmetry introduced by varying the substrate refractive index and can be flexibly tuned via structural parameters – validated by simulations of nonreciprocal responses with varying plate thickness alone. Additionally, Rabi splitting from varying hole depth induces abrupt electromagnetic asymmetry changes in the strong coupling region, offering a new NRIR tuning freedom. This design strategy provides fresh insights for nonreciprocal device research, with the structure holding promise for sensing and nonlinear applications.

## 1 Introduction

Nonreciprocal electromagnetic devices are essential components in modern photonics for controlling light flow and achieving asymmetric transmission. According to Lorentz reciprocity [1], in systems where permittivity and permeability are symmetric, time-invariant, and linear tensors, the transmission characteristics are identical for forward and backward directions. Tailoring these properties of material parameter tensors thus becomes a means to realize nonreciprocal transmission, such as using magneto-optical materials with DC magnetic bias [2], [3], [4], time-varying materials with modulated refractive indices [5], and nonlinear materials whose refractive index changes with local field intensity [6]. Among these, nonlinearity-induced nonreciprocal transmission features passivity and bias-free operation [7], and its combination with metasurfaces offers the prospect of realizing compact and miniaturized nonreciprocal devices in free-space optical paths [6]. However, traditional materials like silicon have relatively small high-order nonlinear coefficients. To generate significant nonlinear effects under low-power pumping, one approach is to combine them with other materials [8], [9], and the other approach requires high-quality-factor cavities to enhance the local field intensity [6], [10]. Notably, bound states in the continuum (BICs), which exhibit strong confinement of local electromagnetic fields [7], [11], [12], provide a favorable platform for realizing nonlinearity-induced nonreciprocal transmission in traditional materials.

BICs can be briefly categorized into different types, such as symmetry-protected BICs (SP-BICs), accidental BICs, and Friedrich–Wintgen BICs (FW-BICs) [7], [13], [14], [15], [16], [17], [18]. By employing various tuning mechanisms, the quality factor of BICs can be reduced to a finite value, forming quasi-bound states in the continuum (QBICs). As a kind of resonant mode with high quality factors, QBICs have boosted a lot of interesting applications, such as in lasers [11], [19], [20], modulators [21], biosensors [22], [23], [24], [25], optical vortex generators [26], and nonlinear optical devices [27], [28], [29], [30], [31], [32], [33]. Compared to other types, for the well-known SP-BICs, external excitation of QBICs can be easily achieved by breaking the inherent symmetry of the structure [14], [34], [35]. By embedding nonlinear effects where the refractive index varies with local field intensity, such as optical Kerr nonlinearity and thermo-optic effects, the localized fields of QBIC modes in the structure induce refractive index changes, causing redshift of the resonant wavelength [6], [36]. Due to the electromagnetic asymmetry in the vertical direction, the structure exhibits nonreciprocal responses to excitations from the upper and lower ports.

Various methods have been developed to tune the nonreciprocal intensity range (NRIR), such as using zero-refractive-index materials [37] or adjusting the ratio of etching groove depth to structural height [6], [10], [38]. However, these designs have room for improvement, e.g., incident angle dependence [37], polarization sensitivity [6], or multiple etching steps [10], [38]. In contrast, we employ a design requiring only single-step etching, with the metasurface unit cell being a plate-hole tetramer with scaled lattice constants. Under normal incidence, the QBIC exhibits polarization-insensitive excitation, with the scattering power dominated by the common term of magnetic dipoles and magnetic toroidal dipoles, as well as the common term of electric quadrupoles and electric toroidal quadrupoles – rendering the vertical field distribution sensitive to parametric variations. Based on this, the electromagnetic asymmetry in the vertical direction (introduced by varying the substrate refractive index) can be flexibly tuned via structural parameters (e.g., plate thickness and hole radius). Embedding Kerr nonlinearity, simulations of nonreciprocal responses for structures with different plate thicknesses validate the flexibility of NRIR tuning. Additionally, the Rabi splitting effect induced by varying hole depth causes abrupt changes in electromagnetic asymmetry within the strong coupling region, providing a new degree of freedom for NRIR regulation. Leveraging the steep edges between peaks and valleys in the Fano-line-shaped transmission spectra, a balance between NRIR and isolation is more easily achieved, with the relative positions of peaks and valleys tunable via structural parameters. The design strategy in this work provides new insights for the research on nonlinearity-induced nonreciprocal devices.

## 2 Structure design and simulation methodology

**Figure 1:**
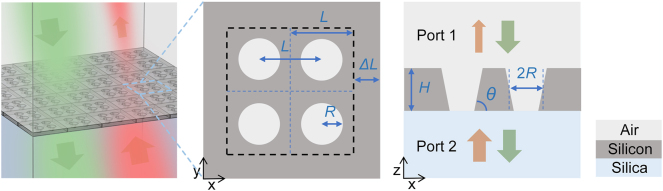
Schematic of the designed metasurface. The unit cell is a plate-hole tetramer with a scaled lattice constant. The pumping light is incident normally from the upper and lower ports, respectively.

The unit cell of the designed metasurface in this work is a tetramer composed of four plate-hole structures (Figure 1), where the perturbation is introduced by scaling the lattice constants of the unit cell. The parameters of the metasurface are labeled as plate thickness *H*, hole radius *R*, hole spacing *L*, sidewall angle *ϑ*, lattice constant 2*L*+2Δ*L*, and the change in lattice constant 2Δ*L* along the *x* or *y* direction. The unit cell is composed of silicon with air holes, overlying air, and a silica substrate. The refractive index of air is taken as 1, that of silica as 1.46, and the refractive index of silicon is referenced from [39]. The pump light is normally incident on the metasurface from the upper or lower port, forming a two-port system overall.

The eigenmodes and transmission spectra of the designed structure are simulated using the finite element method in COMSOL Multiphysics. In the frequency domain of electromagnetic fields, periodic boundary conditions are set in the horizontal direction of the unit cell, while a combination of perfectly matched layers (PML) and scattering boundary conditions is applied in the vertical direction, with sufficient spacing from the unit cell center to ensure the correctness of eigenmode quality factor (Q-factor) calculations. By embedding Kerr nonlinearity in the metasurface structure, nonreciprocal responses are obtained by setting excitation lights with varying power densities at the upper and lower ports. In the nonlinear response, the total polarizability is expressed as $P_{\mathrm{tot}}\left(\omega \right)=\varepsilon_{0}\chi_{\mathrm{eff}}E\left(\omega \right)=\varepsilon_{0}\chi^{\left(1\right)}E\left(\omega \right)+3\varepsilon_{0}\chi^{\left(3\right)}|E\left(\omega \right){|}^{2}E\left(\omega \right)$ , where *χ* ^(1)^ is the linear susceptibility, *χ* ^(3)^ is the third-order nonlinear susceptibility related to the Kerr nonlinearity in this work, and their values are referenced from [40].

## 3 Formation and characteristics of targeted QBICs

**Figure 2:**
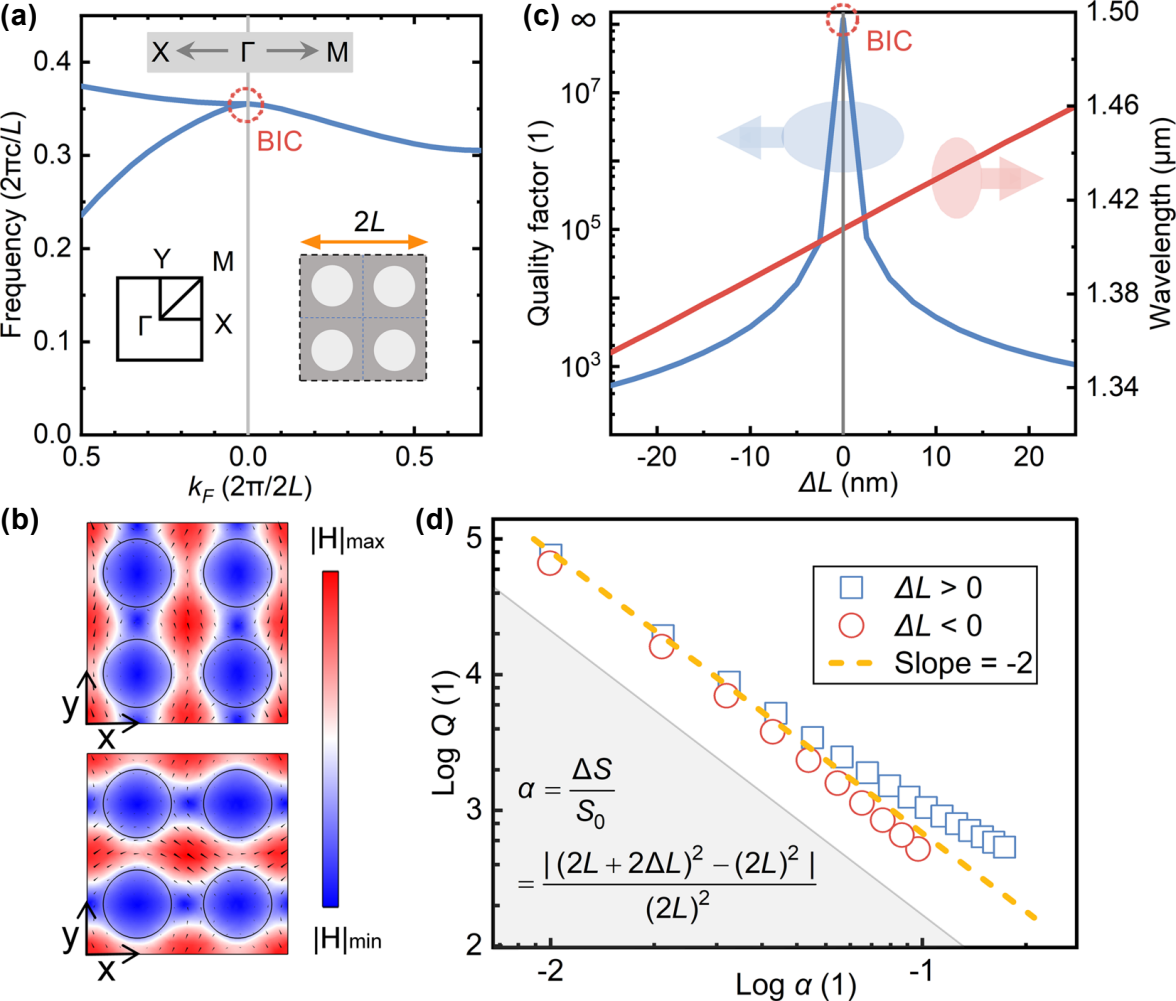
BICs/QBICs eigenmodes in air–substrate plate-hole tetramer unit cell. (a) Band structure at Δ*L* = 0 (BICs at Γ point); inset: Brillouin zone high-symmetry points and metasurface unit cell. (b) *H*-field distributions of orthogonal BIC modes at Γ (arrows: field directions). (c) Q-factor and resonant wavelength versus Δ*L*. (d) Logarithmic QBIC Q-factor versus asymmetry parameter α. Structural parameters: *R* = 170 nm, *L* = 500 nm, *H* = 220 nm, and *ϑ* = 90°.

First, we analyzed the formation process and characteristics of the targeted QBICs. Figure 2a shows the band structure of the BIC mode utilized in this work. The unit cell of the metasurface is an unperturbed plate-hole tetramer suspended in air (inset on the right of Figure 2a), with the following structural parameters: *R* = 170 nm, *L* = 500 nm, *H* = 220 nm, and *ϑ* = 90°. The state at the Γ point in the Brillouin zone originates from band folding caused by the transformation of the unit cell from a single plate-hole structure to a tetramer. During this process, states at the X and Y points fold to the Γ point, forming degenerate states. Away from the Γ point along the ΓX or ΓY direction in the k-space, the Γ-point state splits into two bands. Figure 2b shows the magnetic field distributions of two mutually orthogonal eigenstates, with the *x*–*y* cross-section at the center of the unit cell. In the plate-hole structure without perturbation (Δ*L* = 0), the BIC mode is completely bound within the structure due to structural symmetry protection, without coupling to the radiation background, exhibiting an infinite Q-factor, as shown in Figure 2c. By scaling the lattice constant of the tetramer unit cell, the translational symmetry of the single plate-hole structure is broken, opening the radiation channel of the original bound state, reducing the Q-factor to a finite value, and forming QBICs. Additionally, scaling the lattice constant larger or smaller causes the resonant wavelength to redshift or blueshift, respectively.

Figure 2d describes the relationship between the asymmetry parameter and the Q-factor of QBICs, where the asymmetry parameter is defined as the ratio of the area change of the tetramer unit cell caused by scaling the lattice constant to the initial area. The slope of the logarithmic curve in the figure is close to the theoretical value of −2 in Ref. [14]. For symmetry-protected BICs, different defect positions lead to different degrees of perturbation to the eigenfield [41], [42]. In the tetramer unit cell, even when the asymmetry parameter is the same, the perturbations of the boundary to the eigenfield differ between scaling the lattice constant larger or smaller. Reducing the lattice constant brings the strong-field regions closer, resulting in stronger perturbation to the eigenfield, whereas increasing the lattice constant has the opposite effect. Therefore, in Figure 2d, when the asymmetry parameter is large, the slope of the logarithmic curve between the Q-factor and the asymmetry parameter deviates from −2.

**Figure 3:**
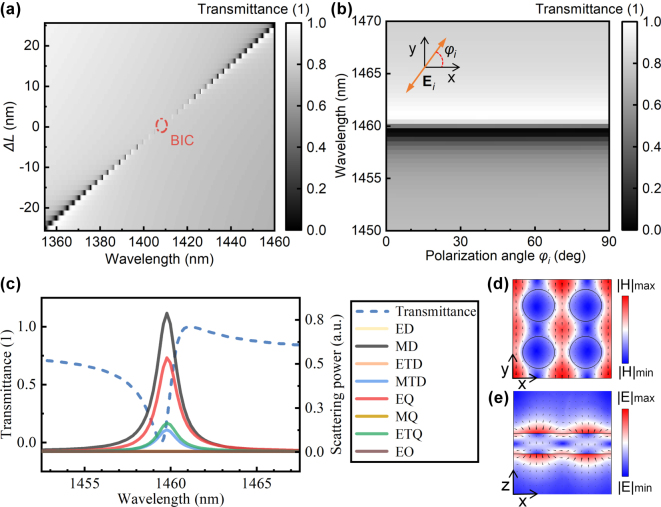
Linear transmission response of air–substrate structure under *z*-direction linearly polarized light. (a) Variation of the transmission spectrum with Δ*L*. (b) Transmission spectra under different polarized excitations, where Δ*L* = 25 nm. (c) Fano-line-shaped transmission spectrum and multipole scattering power (Δ*L* = 25 nm, *Φi* = 0°); multipoles: electric dipole (ED), magnetic dipole (MD), electric toroidal dipole (ETD), magnetic toroidal dipole (MTD), electric quadrupole (EQ), magnetic quadrupole (MQ), electric toroidal quadrupole (ETQ), and electric octupole (EO). (d) *H*-field and (e) *E*-field distributions on the *x*–*y* and *x*–*z* cross-sections of the unit cell center, respectively, at 1,459.6 nm (from (c)), with arrows indicating field directions.

The linear transmission responses of the designed structure were simulated (Figure 3) with the same parameters as in Figure 2. Under excitation with *x*-direction electric field vectors, the transmission spectra of structures with different perturbations are shown in Figure 3a. When Δ*L* = 0, the peak in the transmission spectrum disappears, and the resonant mode decouples from the radiation background, becoming a bound state in the continuum. Furthermore, responses under excitations with different polarization angles were considered (Figure 3b) when Δ*L* = 25 nm. As the electric field azimuth angle varies from 0° to 90°, the transmission peak in the spectrum hardly shifts, demonstrating that the designed QBIC mode is polarization-insensitive. This phenomenon is attributed to the structural characteristics: the tetramer unit cell maintains π/4 rotational symmetry while the perturbation disrupts the translational symmetry of the single plate-hole structure.

Multipole decomposition was used to analyze the scattering power under the polarization angle of 0° in Figure 3b, with the calculation method detailed in Supplementary Material S1. In the scattering power of a single multipole, the magnetic dipole (MD) and electric quadrupole (EQ) make the largest contributions, while the contributions of the magnetic toroidal dipole (MTD) and electric toroidal quadrupole (ETQ) cannot be neglected either (Figure 3c). In fact, under the sub-diffraction limit, multipole decomposition of the scattered field reveals that the terms of MD & EQ and MTD & ETQ form destructive interference, respectively. The QBIC mode mainly originates from the combined effect of these four components (see Supplementary Material S1 for detailed analysis). Figure 3d and e show the magnetic and electric field distributions in the structure at a wavelength of 1,459.6 nm, with arrows indicating the directions of the magnetic and electric fields. The cross-sections are the *x*–*y* plane and *x*–*z* plane at the center of the unit cell, respectively. As can be seen from Figure 3e, the target mode forms a leaky electric field distribution at the upper and lower surfaces, which makes the mode field susceptible to changes in the refractive index of the substrate. According to the variation principle of energy, the mode center tends to shift toward the side with a higher refractive index.

## 4 Influence of substrate refractive indices and structural parameters on QBICs

**Figure 4:**
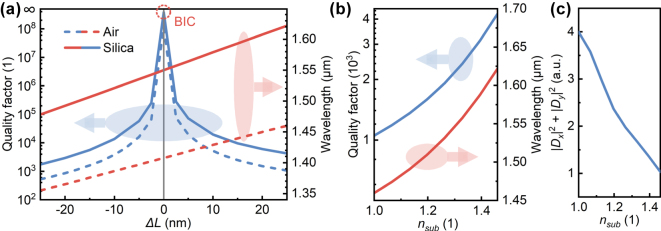
Effect of substrate refractive index on QBICs. (a) Variations of the QBIC quality factor and resonant wavelength with ΔL, where the substrates are air and silica, respectively. (b) Variations of the QBICs quality factor and resonant wavelength with the substrate refractive index at Δ*L* = 25 nm. (c) Coupling amplitude from the resonant mode to two orthogonal zero-order diffraction channels versus the substrate refractive index.

The influence of changing the substrate refractive index on the Q-factors and resonant wavelengths of QBICs was investigated (Figure 4). After replacing the air substrate in Figure 2 with silica, when there is no perturbation (Δ*L* = 0), the resonant mode remains a BIC with an infinite Q-factor, but the resonant wavelength redshifts from 1,408 nm to 1,550 nm (Figure 4a). When a perturbation is introduced (Δ*L* ≠ 0), changing the substrate material significantly alters both the Q-factor and resonant wavelength of QBICs. Figure 4b calculates the variations of QBIC Q-factors and resonant wavelengths with the substrate refractive index at Δ*L* = 25 nm. It can be seen that as the substrate refractive index increases, the resonant wavelength of QBICs redshifts, consistent with the point of BICs, while the Q-factor of QBICs increases by a certain margin. To analyze the cause, the coupling amplitudes (*D*_*x*(*y*)_) between QBICs and two orthogonal zero-order diffraction channels were calculated, with the calculation method provided in Supplementary Material S2. As the substrate refractive index increases, |*D*_*x*_|^2^ + |*D*_*y*_|^2^ decreases (Figure 4c). Since *γ*/*c* = |*D*_*x*_|^2^ + |*D*_*y*_|^2^, this implies that the total decay rate of the QBIC mode decreases when the substrate refractive index increases from 1 to 1.46; thus, the structure with a silica substrate exhibits a higher Q-factor.

**Figure 5:**
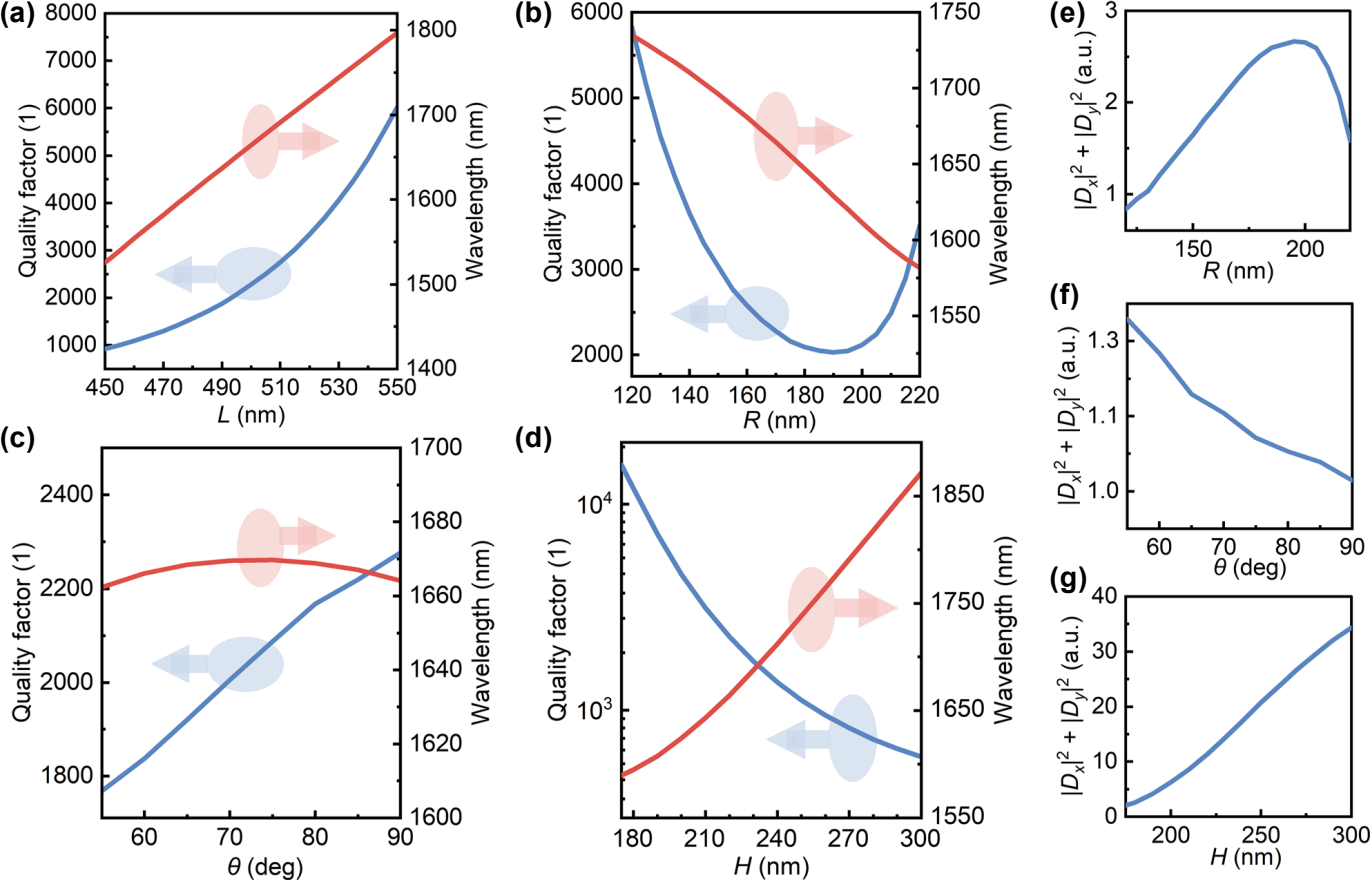
Quality factor and resonant wavelength of QBICs as a function of structural parameters. The structure has a silica substrate with Δ*L* = 40 nm. The adjusted structural parameters are (a) hole spacing *L*, (b) hole radius *R*, (c) sidewall angle *ϑ*, and (d) plate thickness *H*. Variations of the coupling amplitude from the resonant mode to two orthogonal zero-order diffraction channels with (e) hole radius, (f) sidewall angle, and (g) plate thickness.

The effects of other structural parameters on QBICs were analyzed (Figure 5). The structure used a silica substrate, with Δ*L* set to 40 nm. Other parameters were kept consistent with those in Figure 2, and only one parameter was altered during calculations. As shown in Figure 5a–d, adjusting the hole spacing, hole radius, sidewall angle, or plate thickness all affect the Q-factor and resonant wavelength. An increase in *L* reduces the asymmetry parameter *α*, thereby boosting the Q-factor. Although tuning *R*, *ϑ*, or *H* does not alter the asymmetry parameter *α*, it modifies the eigenfield distribution within the structure. Consequently, even with constant Δ*L*, the perturbations to the eigenfield differ, leading to variations in the resonant mode’s Q-factor. Figure 5e–g depict the coupling amplitudes from the resonant mode to two orthogonal zero-order diffraction channels for *R*, *ϑ*, and *H* adjustments, respectively. A peak decay rate occurs at *R* = 193 nm, where the eigenfield experiences maximal perturbation, corresponding to the Q-factor minimum in Figure 5b. When the sidewall angle decreases from 90° to 55°, the coupling amplitude shows only minor variations (Figure 5f), whereas increasing the plate thickness from 175 nm to 300 nm induces more pronounced variations in the coupling amplitude (Figure 5g). This demonstrates that the QBIC eigenfield distribution is more sensitive to the plate thickness than the sidewall angle.

**Figure 6:**
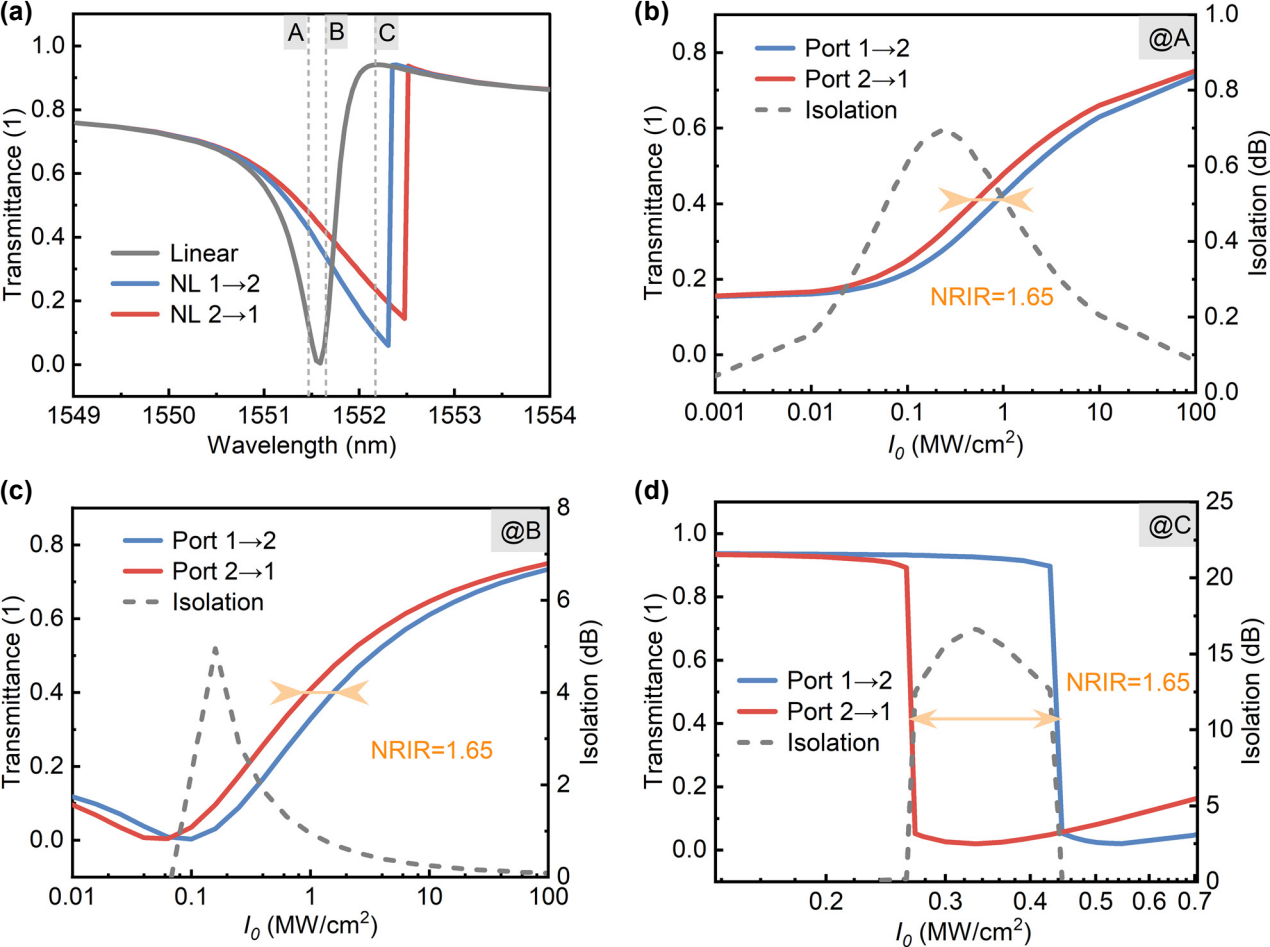
Nonreciprocal responses of the same structure at different pumping wavelengths. (a) Linear transmission spectrum (gray solid line) versus that with Kerr nonlinearity. (b)–(d) Variations of the nonreciprocal response of the metasurface at three pumping wavelengths in (a) with the incident light power density. The blue and red solid lines in (a)–(d) represent the transmittances from Port 1 to Port 2 and the reverse direction, respectively. The gray dashed line in (b)–(d) represents the isolation degree. The structural parameters are *R* = 170 nm, *L* = 500 nm, *H* = 240 nm, Δ*L* = −30 nm, ϑ = 90°, and the substrate is silica.

Among all parameters, the sidewall angle exerts the slightest influence on the resonant wavelength: varying *ϑ* from 90° to 55° results in only a few nanometers of wavelength shift. By comparison, expanding the hole spacing or plate thickness, and reducing the hole radius, can trigger redshift of the resonant wavelength by tens to hundreds of nanometers. The resonant wavelength can be flexibly tuned by adjusting structural parameters. For instance, when *H* is changed from 220 nm to 240 nm and Δ*L* from 40 nm to −30 nm, the resonant wavelength can be shifted to around 1,550 nm (gray solid line in Figure 6a). These features establish a foundation for tuning the relative positions of peaks and valleys in the Fano-line-shaped transmission spectra, as detailed in Supplementary Material S3.

## 5 Nonreciprocal transmission based on QBICs with Kerr nonlinearity

The characteristics of nonreciprocal transmission based on QBIC modes were investigated by embedding Kerr nonlinearity in the designed structure. The structure with a silica substrate was adopted, featuring the following parameters: *R* = 170 nm, *L* = 500 nm, *H* = 240 nm, Δ*L* = −30 nm, and *ϑ* = 90°. Linearly polarized light with a power density of 1 MW/cm^2^ was incident from Port 1 and Port 2, respectively. Compared to the linear transmission spectrum, the resonant wavelengths in the transmission spectra exhibited redshift with different magnitudes (Figure 6a). In the Kerr effect, the refractive index change is related to the square of the local electric field strength, expressed as *n* = *n*_0_ + Δ*n* = *n*_0_ + *n*_2_|*E*|^2^, where *n*_2_ is the nonlinear coefficient of the Kerr effect. The QBIC modes of high Q-factors confine the optical field within the structure with a low decay rate. Due to the nonlinear term, the refractive index increases at strong local field positions, causing redshift of the resonant wavelength. Furthermore, the vertical asymmetry induced by substituting the substrate with silica leads to unequal electric field intensities in the excited resonant modes under identical pump power density at both ports, thus yielding different wavelength shifts for the two excitation directions.

The nonreciprocal responses of the same structure to different excitation wavelengths were studied (Figure 6b–d). The NRIR is defined as $Max\left(I_{1}/I_{2},I_{2}/I_{2}\right)$ , where *I*_1_ and *I*_2_ are the pump power densities at Port 1 and Port 2 for the same transmittance. The excitation wavelengths in Figure 6b–d correspond to the wavelengths at points A–C in Figure 6a. It can be seen that in all three cases, NRIR is equal to 1.65. According to the coupled mode theory for two-port systems, NRIR can be represented by the asymmetry parameter *κ* in the linear regime [36], [43], i.e., $NRIR=Max\left(\kappa ,1/\kappa \right)=Max\left(\gamma_{1}/\gamma_{2},\gamma_{2}/\gamma_{1}\right)$ , where *γ*_1_ and *γ*_2_ are the decay rates of the resonant mode to the two ports. Since the decay rate *γ* depends only on structural parameters, this explains why the same structure exhibits identical NRIR for different excitation wavelengths.

The isolation is defined as $10\text{Log}\left[Max{\left(T_{1}/T_{2},T_{2}/T\right)}_{1}\right]$ , where *T*_1_ and *T*_2_ denote transmittances for Port 1 and Port 2 excitations at identical pump power. As shown in Figure 6b–d, Fano-line peak-wavelength excitation yields higher isolation (up to >15 dB) than valley-wavelength excitation. In nonreciprocal transmission, increasing pump power density induces resonant wavelength redshift. The Fano line’s asymmetric profile (left valley, right peak) dictates distinct transmittance behaviors: as the pump power increases, peak excitation follows a steep descending edge, while valley excitation follows a gentle ascending edge. Consequently, for the same NRIR, peak-wavelength excitation enables greater isolation. To achieve a steep ascending edge at valley excitation, tuning the Fano-line peak-valley relative positions is essential. As mentioned before, the Fano line originates from the coupling between a narrow FWHM resonant mode and a broad background mode (ED). Therefore, the relative positions of the peak and valley in the Fano line can be adjusted by tuning the relative magnitudes of the central frequencies of the two modes through structural parameters, as elaborated in Supplementary Material S3.

According to $NRIR=Max\left(\kappa ,1/\kappa \right)$ , the relationship between NRIR and structural parameters can be transformed into the relationship between electromagnetic asymmetry and structural parameters in the linear regime. Now consider the structure excited by only the *i*th port, with the signal amplitude *E*_*i*_ and frequency *ω*. According to coupled mode theory [44], [45], the amplitude of the resonance is given by $a_{i}=k_{i}E_{i}/\left[i\left(\omega -\omega_{0}\right)+\gamma \right]$ , where *k*_*i*_ is the coupling coefficient between the *i*th port and the resonator, related to the decay rate by 2*γ*_*i*_ = |*k*_*i*_|^2^. Thus, electromagnetic asymmetry is expressed as $\kappa =\gamma_{1}/\gamma_{2}=|k_{1}{|}^{2}/|k_{2}{|}^{2}=|a_{1}{|}^{2}|E_{2}{|}^{2}/\left(|E_{1}{|}^{2}|a_{2}{|}^{2}\right)$ , meaning that when the power densities and wavelengths of the two port excitations are identical, *κ* can be obtained from the ratio of light intensities in the structure. In the following calculations, the amplitude of the resonance in the structure is taken as the average of the electric field norm in the silicon of the unit cell.

**Figure 7:**
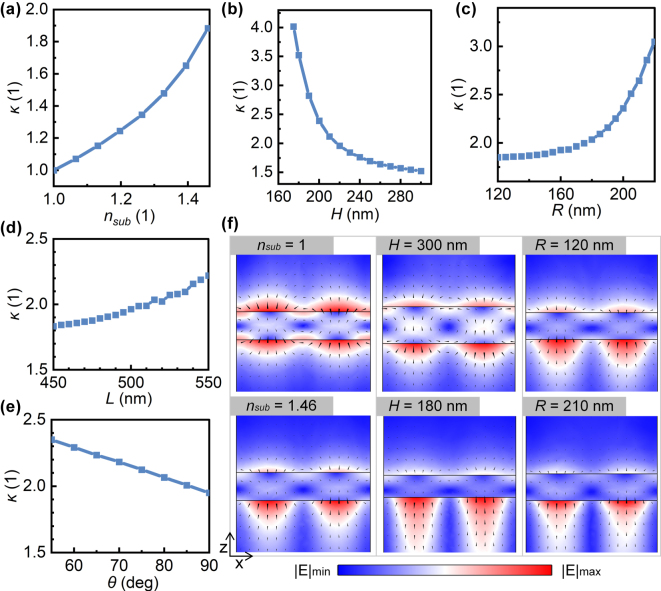
Variation of electromagnetic asymmetry in the linear regime with substrate refractive indices and structural parameters. The parameters are Δ*L* = 40 nm, *n*_sub_ = 1.46, *H* = 220 nm, *R* = 170 nm, *L* = 500 nm, and *ϑ* = 90°. Only one parameter is varied at a time, which are (a) substrate refractive index, (b) plate thickness, (c) hole radius, (d) hole spacing, and (e) sidewall angle. (f) *E*-field distributions on the *x*–*y* cross-sections of the unit cell center, with arrows indicating field directions.

Figure 7 illustrates the effects of different substrate refractive indices and structural parameters on electromagnetic asymmetry *κ*. The parameters here are consistent with those in Figure 5, which are Δ*L* = 40 nm, *n*_sub_ = 1.46, *H* = 220 nm, *R* = 170 nm, *L* = 500 nm, and *ϑ* = 90°, with only one parameter modified each time. As shown in Figure 7a, when *n*_sub_ = 1, *κ* = 1, indicating identical resonance amplitudes excited by the upper and lower ports. In this case, the electric field exhibits perfect symmetry in the vertical cross-section (the *n*_sub_ = 1 panel in Figure 7f). Increasing *n*_sub_ enhances *κ* and shifts the mode center toward the substrate (the *n*_sub_ = 1.46 panel in Figure 7f). Hence, NRIR originates from substrate-induced electromagnetic asymmetry. Among structural parameters, tuning the plate height and hole radius markedly alters *κ*. An *H* decrease from 300 nm to 175 nm boosts *κ* by ∼2.5, while *R* increasing from 120 nm to 220 nm raises it by ∼1.2. Conversely, vertical parameters like sidewall angle show weak effects: *ϑ* decreasing from 90° to 55° increases *κ* by ∼0.4, and hole spacing changes (450–550 nm) yield <0.4 *κ* growth. Comparing field distributions in Figure 7f, mode centers shift vertically for different *H*/*R*, consistent with the trend caused by changing the substrate refractive index. These variations can be attributed to the multipole composition of the target optical mode, which renders the vertical field distribution susceptible to parameter changes. By analyzing the changes in multipole contributions caused by variations in the substrate refractive index and plate thickness, the results indicate that the asymmetry of the vertical field distribution originates from the influence of additional multipole terms (electric toroidal dipole (ETD), magnetic quadrupole (MQ) and electric octupole (EO)) introduced in the scattered field. See Supplementary Material S4 for a detailed analysis.

**Figure 8:**
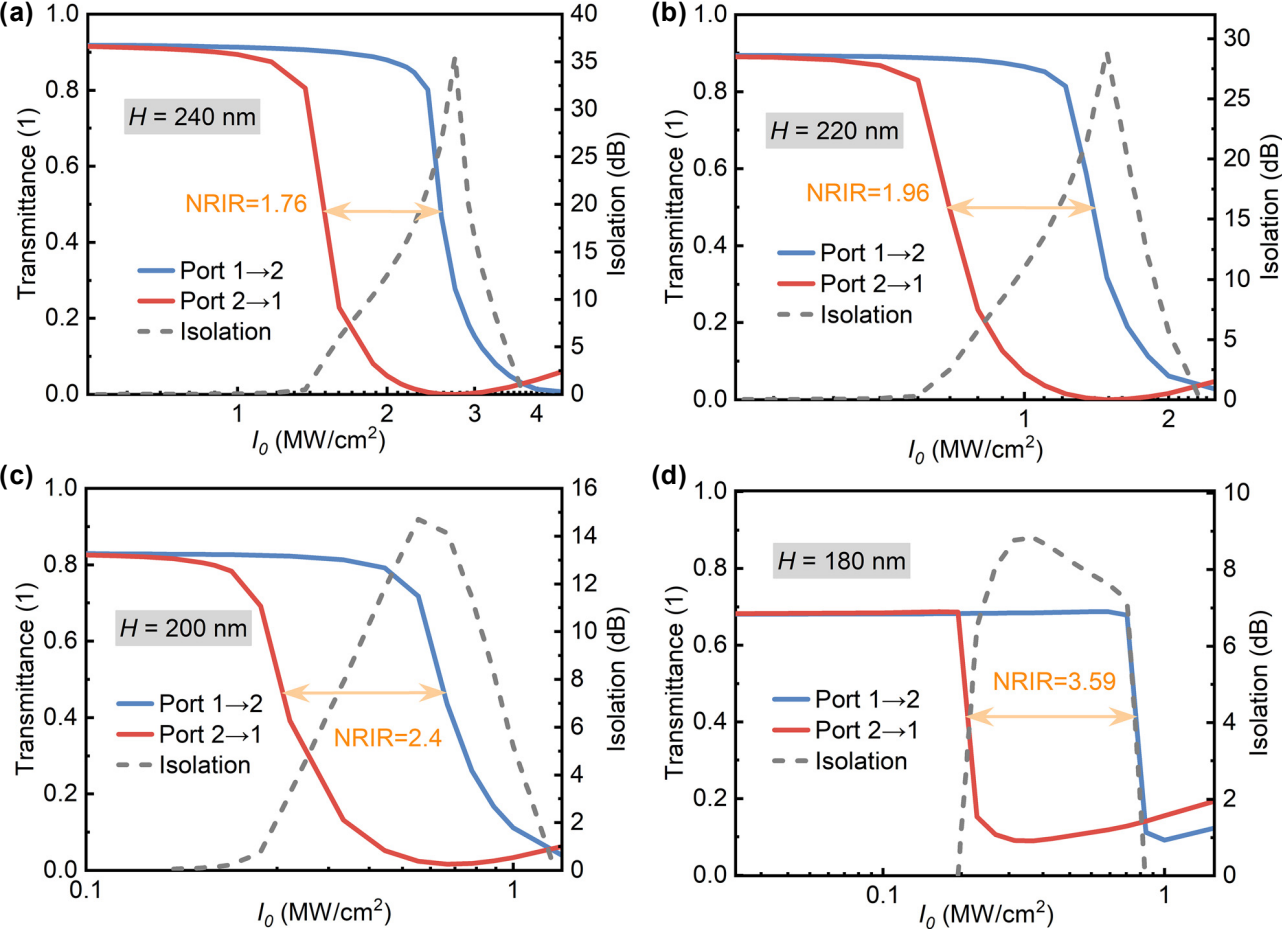
Nonreciprocal responses of metasurfaces with varying plate thicknesses: (a) 240 nm, (b) 220 nm, (c) 200 nm, and (d) 180 nm, with corresponding pumping wavelengths of 1,713.34 nm, 1,664.59 nm, 1,624.08 nm, and 1,594.97 nm, respectively.

To verify the influence of electromagnetic asymmetry changes in the linear regime on nonreciprocal transmission, nonreciprocal responses of structures with different plate thicknesses to variable power density excitations at two ports were further calculated (Figure 8), where the pump light wavelength was selected near the Fano-line peak. When the plate thicknesses are 240 nm, 220 nm, 200 nm, and 180 nm, NRIR values are 1.76, 1.96, 2.4, and 3.59, respectively, consistent with the calculations of *κ* in Figure 7b. The transmittance and NRIR are related by *T*_max_ ≤ 4NRIR/(NRIR^2^ + 1) [36]. Increasing NRIR reduces the maximum transmittance, in agreement with the results in Figure 8, indicating a trade-off between NRIR and transmittance. Additionally, thinner plates lead to lower power density at the minimum transmittance: as the plate thickness decreases from 240 nm to 180 nm, this power density drops from about 3 MW/cm^2^ to less than 0.3 MW/cm^2^. This is because reducing the plate thickness increases the Q-factor of QBICs (Figure 3d), enhancing light–matter interaction. The normalized power is proportional to the square of the Q-factor [43], meaning a higher Q-factor implies higher energy utilization efficiency.

**Figure 9:**
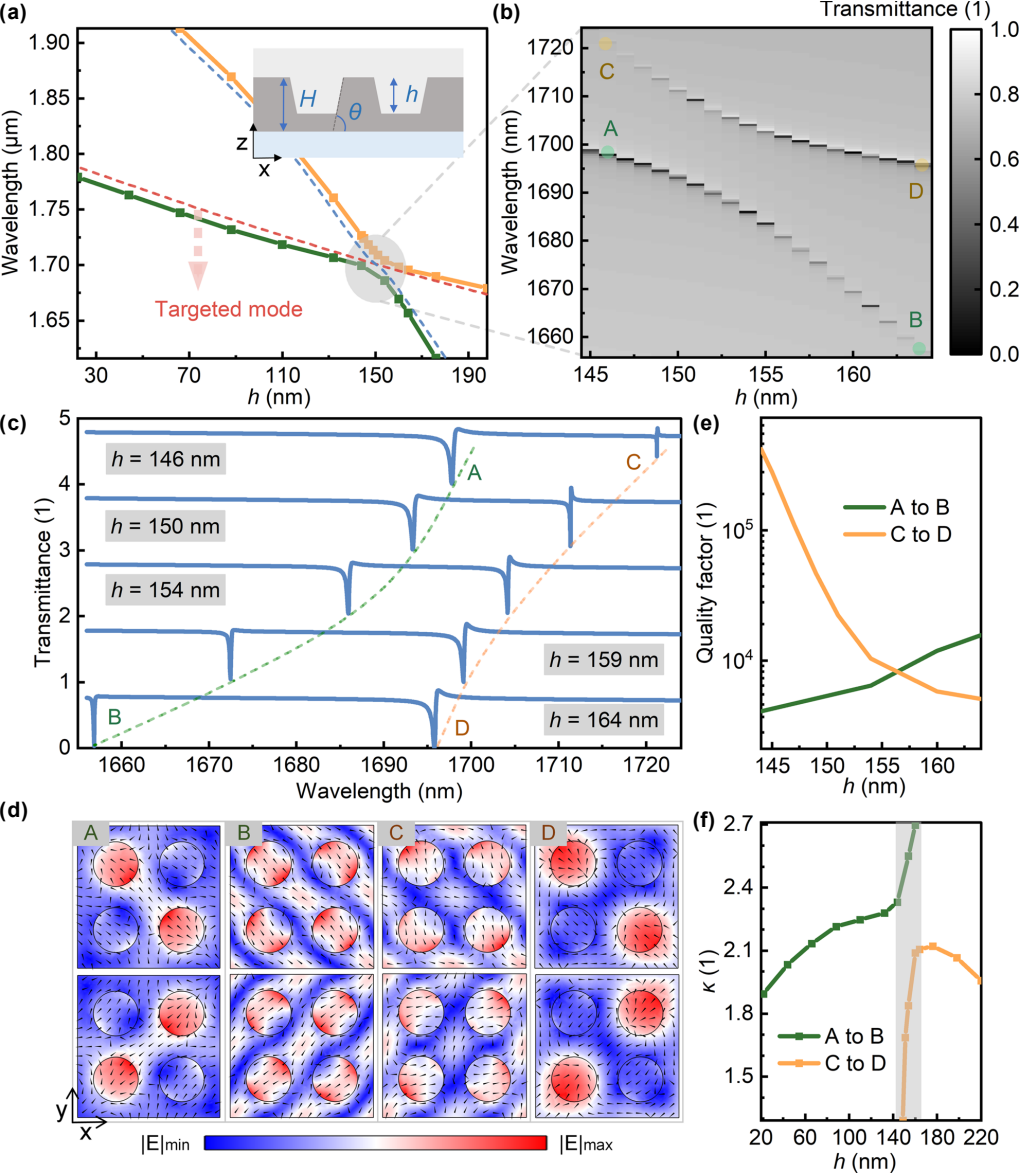
Rabi splitting and electromagnetic asymmetry versus hole depth. (a) Resonant wavelengths of two modes versus the hole depth (inset: definition of the hole depth *h* in the unit cell). (b) Transmission spectra and (c) line shapes at the crossing region. (d) Eigenfields from A to D (*x*–*y* cross-section at unit cell center; arrows: electric field vectors). (e) Quality factors of eigenmodes on both bands in the crossing region. (f) Electromagnetic asymmetry on both bands versus the hole depth.

In addition to the parameters mentioned earlier, the effect of hole depth *h* on the electromagnetic asymmetry of the target optical mode was studied, with the definition of hole depth shown in the inset of Figure 9a. Decreasing the hole depth causes the resonant wavelengths of the target optical mode and another mode to redshift simultaneously, with a crossing region near *h* = 154 nm (Figure 9a). Figure 9b and c show the transmission spectra and line shape changes in the crossing region, indicating that the two bands do not intersect. By calculating the eigenmodes on the two bands, it is found that the modes from point A to B and point C to D both change (Figure 9d), with the Q-factor decreasing from A to B and increasing from C to D (Figure 9e). Thus, it can be inferred that in the crossing region, the target optical mode strongly couples with another mode, producing the Rabi splitting effect, where the energy transfer rate between the two modes exceeds the decay rate. Further analysis of electromagnetic asymmetry across the two bands uncovers an abrupt transition in the crossing region (Figure 9f), attributed to the influence of an additional optical mode. Multipole decomposition analysis reveals that the abrupt change from point B to point C results from two causes: First, the increased contributions of the ETD, MQ and EO give rise to the asymmetry of the vertical field distribution (this corresponds to point B); second, when the contribution ratios of all multipoles are comparable, destructive interference occurs, leading to an improved quality factor (this corresponds to point C). At this point (i.e., point C), the electric field distribution is primarily along the horizontal direction (see Fig. S5(c)). For a detailed analysis, refer to Supplementary Material S5. The Rabi splitting effect in the crossing region provides a new degree of freedom for tuning NRIR.

## 6 Conclusions

In summary, we analyzed the characteristics of QBICs realized by scaling lattice constants, focusing on their nonlinearity-induced nonreciprocal transmission. The target BIC mode originates from band folding in the Brillouin zone, and perturbation caused by scaled lattice constants transforms BICs into QBICs. The unit cell of the metasurface is a plate-hole tetramer requiring only single-step etching, and its π/4 rotational symmetry enables polarization-insensitive excitation of QBICs under normal incidence. Multipole decomposition reveals that the scattering power of QBIC modes primarily stems from the common term of magnetic dipoles and magnetic toroidal dipoles, as well as the common term of electric quadrupoles and electric toroidal quadrupoles – rendering the vertical field distribution sensitive to parametric variations. Increasing the substrate refractive index redshifts the QBIC resonant wavelength and enhances its quality factor, both of which can be flexibly tuned via structural parameters.

By embedding Kerr nonlinearity, nonreciprocal responses of the same structure to different excitation wavelengths demonstrate that leveraging the steep edge between the peak and valley of the Fano-line shape in transmission spectra facilitates balancing the NRIR and isolation. The relative peak-valley positions are tunable by adjusting the relative central frequencies of the target optical mode and background mode. The NRIR of the designed structure originates from vertical electromagnetic asymmetry introduced by changing the substrate refractive index, which can be flexibly tuned by merely reducing the metasurface plate thickness. Expanding the hole radius or spacing and decreasing the sidewall angle also enhance the electromagnetic asymmetry. Leveraging the property that the vertical field distribution shifts toward the side of the substrate or overlay with a higher refractive index, replacing this side with other nonlinear materials [8], [9] is expected to further reduce the power required for nonreciprocal behavior. Additionally, the Rabi splitting effect induced by changing the hole depth causes abrupt changes in electromagnetic asymmetry within the strong coupling region, providing a new degree of freedom for NRIR regulation. The structure in this work holds potential for further applications in sensing and nonlinear fields, and the design strategy offers novel insights for research on nonreciprocal devices.

## Supplementary Material

Supplementary Material Details
