# Supplementary material for: Nonreciprocal transmission based on quasi-bound states in the continuum via scaled lattice constants
Source: Nanophotonics. 2025 Sep 30;14(22):3611–23. doi: 10.1515/nanoph-2025-0320 (PMC12592790; doi:10.1515/nanoph-2025-0320)
Supplement: Supplementary file 1 — Supplementary Material Details [file j_nanoph-2025-0320_suppl_001.pdf]

Supplementary Material

Nonreciprocal transmission based on quasi-bound states in the continuum via scaled lattice constants

Ran Li and Junqiang Sun*

***Corresponding author: Junqiang Sun**, Huazhong University of Science and Technology, Wuhan, China; jqsun@hust.edu.cn;

Ran Li: Huazhong University of Science and Technology, Wuhan, China; r_li_2021@foxmail.com;

S1 Multipole decomposition

Defining the time-harmonic factor of the incident light as $e^{i\omega t}$, the polarization current density within a unit cell in the frequency domain is given by $\vec{J}(\vec{r}) = \mathrm{i}\omega\varepsilon_0(\varepsilon - \varepsilon_d)\vec{E}(\vec{r})$, where ω denotes the angular frequency, ε_0 is the vacuum permittivity, and ε represents the relative permittivity of the metasurface material, ε_d is the relative permittivity of the background material. The definitions of the multipole moments under Cartesian coordinate [1-3] are as follows,

Electric Dipole (ED) Moment:

$$p_i = \frac{1}{\mathrm{i}\omega}\int J_i \mathrm{d}v$$

Electric Toroidal Dipole (ETD) Moment:

$$T_i^{(e)} = \frac{1}{10}\int \{(\vec{J}\cdot\vec{r})r_i - 2r^2 J_i\}\mathrm{d}v$$

Magnetic Dipole (MD) Moment:

$$m_i = \frac{1}{2}\int (\vec{r}\times\vec{J})_i \mathrm{d}v$$

Magnetic Toroidal Dipole (MTD) Moment:

$$T_i^{(m)} = \frac{-\mathrm{i}\omega}{20}\int r^2(\vec{r}\times\vec{J})_i \mathrm{d}v$$

Electric Quadrupole (EQ) Moment:

$$Q_{ij}^{(e)} = \frac{1}{\mathrm{i}\omega}\int \left\{r_i J_j + r_j J_i - \frac{2}{3}\delta_{ij}(\vec{r}\cdot\vec{J})\right\}\mathrm{d}v$$

Electric Toroidal Quadrupole (ETQ) Moment:

$$T_{ij}^{(Qe)} = \frac{1}{42}\int \begin{Bmatrix} 4(\vec{r}\cdot\vec{J})r_i r_j + 2\delta_{ij}(\vec{r}\cdot\vec{J})r^2 \\ -5r^2(r_i J_j + r_j J_i) \end{Bmatrix}\mathrm{d}v$$

Magnetic Quadrupole (MQ) Moment:

$$Q_{ij}^{(m)} = \frac{1}{3}\int \left\{(\vec{r}\times\vec{J})_i r_j + (\vec{r}\times\vec{J})_j r_i\right\}\mathrm{d}v$$

Electric Octupole (EO) Moment:

$$O_{ijk}^{(e)} = \frac{1}{\mathrm{i}\omega}\int \left\{ \begin{array}{c} r_i r_j J_k + r_j r_k J_i + r_k r_i J_j \\ -\frac{1}{5}\left\{ \begin{array}{c} \delta_{ij}[r^2 J_k + 2r_k(\vec{r}\cdot\vec{J})] \\ +\delta_{jk}[r^2 J_i + 2r_i(\vec{r}\cdot\vec{J})] \\ +\delta_{ki}[r^2 J_j + 2r_j(\vec{r}\cdot\vec{J})] \end{array} \right\} \end{array} \right\}\mathrm{d}v.$$

The total scattering power of all directions for multipole decomposition is given by

$$P_{\text{scat}} = \frac{\sqrt{\varepsilon_d}k_0^4 c}{12\pi\varepsilon_0}\left\{\sum_i\left[\left|p_i - \frac{\mathrm{i}k_d}{v_d}T_i^{(e)}\right|^2 + \frac{1}{v_d^2}\left|m_i - \frac{\mathrm{i}k_d}{v_d}T_i^{(m)}\right|^2\right] + \sum_{i,j}\left[\frac{3k_d^2}{40}\left|Q_{ij}^{(e)} - \frac{\mathrm{i}k_d}{v_d}T_{ij}^{(Qe)}\right|^2 + \frac{3k_d^2}{40v_d^2}\left|Q_{ij}^{(m)}\right|^2\right] + \sum_{i,j,k}\frac{k_d^4}{315}\left|O_{ijk}^{(e)}\right|^2\right\}.$$

In the above equations, subscript i, j, k = x, y, z. k_d and v_d are wave number and light speed in the background material. The scattering field of multipole decomposition with sub-diffraction limit and farfield approximation is given by

$$E_i = \frac{-\mathrm{i}k_0}{2\varepsilon_0\sqrt{\varepsilon_d}S}e^{-\mathrm{i}k_d|z|}\left\{\delta_{ij}\left[\left(p_j - \frac{\mathrm{i}k_d}{v_d}T_j^{(e)}\right) - \frac{\mathrm{i}k_d}{2}\left(Q_{jz}^{(e)} - \frac{\mathrm{i}k_d}{v_d}T_{jz}^{(Qe)}\right) - \frac{k_d^2}{6}O_{jzz}^{(e)}\right] + \epsilon_{ij}\left[-\frac{1}{v_d}\left(m_j - \frac{\mathrm{i}k_d}{v_d}T_j^{(m)}\right) + \frac{\mathrm{i}k_d}{2v_d}Q_{jz}^{(m)}\right]\right\}.$$

Here, δ_{ij} and ϵ_{ij} are Kronecker symbols and Levi-Civita symbols, respectively, with subscript i, j = x, y.

For the structure in Fig. 3(a), when ΔL equals 40 nm, the multipole decomposition of the scattered field, excited by x-polarized light incident along the z-direction, is shown in Fig. S1. Among the components, MDy, EQxz, MTDy, and ETQxz make the primary contributions. By calculating the real and imaginary parts of these components, it can be observed that MDy and EQxz undergo destructive interference, and similarly, MTDy and ETQxz also exhibit destructive interference.

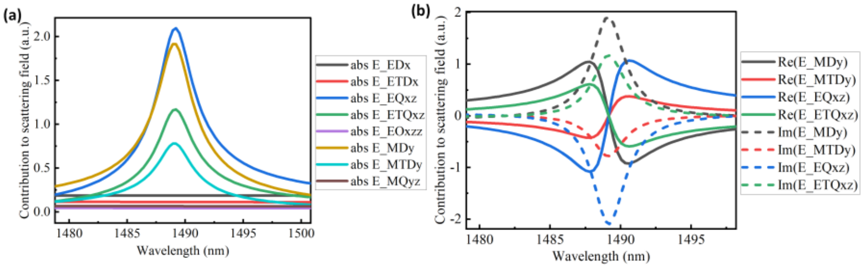

Fig. S1. Multipole decomposition of the scattered field in the structure with an air substrate. (a) Absolute values of the scattered field contribution terms of each multipole. (b) Real and imaginary parts of the scattered field for the magnetic dipole (MD), magnetic toroidal dipole (MTD), electric quadrupole (EQ), and electric toroidal quadrupole (ETQ) terms.

S2 Calculation of D_x and D_y

For QBICs with the frequencies below the diffraction limit, the inverse radiation lifetime γ_{tot} is defined as radiation losses into zeroth-order diffraction channels. The γ_{tot} takes the form [4]:

$$\gamma_{tot} = \frac{S_0 \sum_{z=\pm\infty} \sum_{i=x,y} \left|E_{rs,i}(z)\right|^2}{\int \varepsilon |\boldsymbol{E}_{rs}|^2 \mathrm{d}V} = \sum_{i=x,y} |D_i|^2,$$

$$\left|E_{rs,i}(z \to \pm\infty)\right|^2 = \frac{\omega_0^2}{4S_0^2 c^2} \left|\int (\varepsilon - 1)\, E_{\mathrm{rs},i} e^{\pm i k_0 z'} \mathrm{d}V'\right|^2.$$

D_x and D_y are two orthogonal coupling amplitudes of QBICs to the zeroth-order diffraction channels. S_0, ω_0 and E_{rs} denote the area of the unit cell in x-y plane, the resonant frequency and the electric field of the resonant mode, respectively, where the integral is performed over the unit cell.

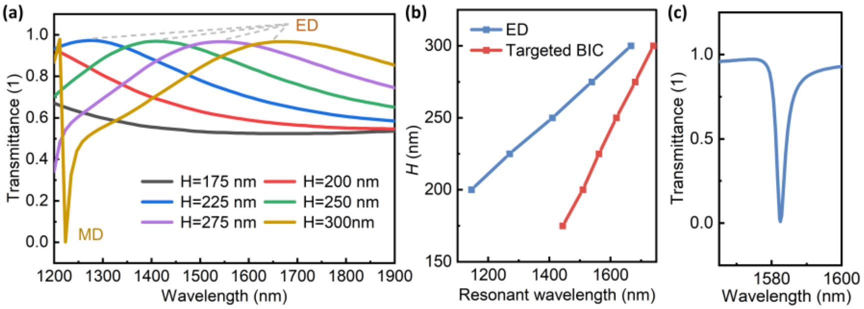

Fig. S2. (a) Transmission spectra of the undisturbed structure under different plate thicknesses. (b) Variation of resonant wavelengths of the Electric Dipole (ED) mode and the designed BIC mode with plate thickness. (c)Fano line shape with left peak and right valley under tuned parameters: R=145 nm, L=450 nm, H=300 nm, θ=90°, ΔL=-25 nm.

S3 Fano resonance in double-oscillator model

In the double-oscillator model [5, 6], Fano resonance is a resonance form where only one oscillator is driven under weak coupling, and the amplitude of the driven oscillators expressed as $|x_1(\Omega)|^2 \approx A\frac{(\Omega+q)^2}{\Omega^2+1}$, where q is the Fano line shape asymmetry factor, defined as q=cot(δ) with $\delta = \mathrm{Arg}[1/(\omega_2 - \omega_1 + i\gamma_1)]$. Theoretically, adjusting the relative magnitude of the

two oscillators' resonant frequencies (ω_1 and ω_2) changes the sign of q, which further alters the relative positions of the peak and valley in the Fano line shape.

In the Fano resonance of the designed structure, the ED mode serves as a broad background mode, whose central wavelength can be widely tuned by changing the metasurface thickness (Fig. S2(a)). Increasing the plate thickness also redshifts the BICs mode resonant wavelength (Fig. S2(b)) but at a slower rate than the ED mode. At *H* = 300 nm, the two central wavelengths are separated by only ~50 nm. Introducing perturbation induces weak coupling between the two modes, forming Fano resonance. According to Fig. 4(a), a negative *ΔL* blueshifts the QBICs resonant wavelength. Reducing *L* also blueshifts the QBICs wavelength (Fig. 5(a)), while decreasing *R* simultaneously could balance the quality factor degradation caused by shortening *L* (Fig. 5(b)). Fig. A.1(c) shows a Fano line shape with a left peak and right valley obtained by tuned parameters: *R* = 145 nm, *L* = 450 nm, *H* = 300 nm, *ϑ* = 90°and *ΔL* = -25 nm.

hape with a left peak and right valley obtained by tuned parameters: *R* = 145 nm, *L* = 450 nm, *H* = 300 nm, *ϑ* = 90°and *ΔL* = -25 nm.

S4 Multipole contributions to vertical field distribution

When the substrate is air, the field in the structure exhibits a symmetric distribution, and the electric toroidal dipole (ETD) and electric octupole (EO) make almost no contribution to the scattering power. However, when the substrate is replaced with silica, the contributions of other terms, such as ETD, EO and MQ increase. Additionally, as the plate thickness decreases, the proportion of contributions from ETD and EO rises (see Fig. S3). From this, it can be concluded that the involvement of ETD and EO terms enhances the asymmetry of the vertical field distribution.

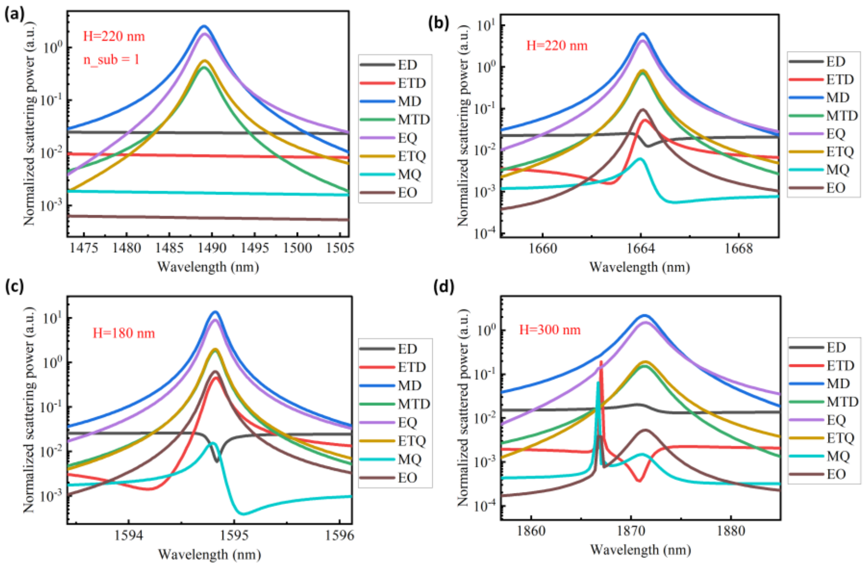

Fig. S3. Multipole decomposition of the scattering power for structures with different substrates and plate thicknesses. (a) Air substrate. (b)–(d) Silica substrate, with plate thicknesses of 220 nm, 180 nm, and 300 nm, respectively.

S5 Abrupt Change in Electromagnetic Asymmetry During Rabi Splitting

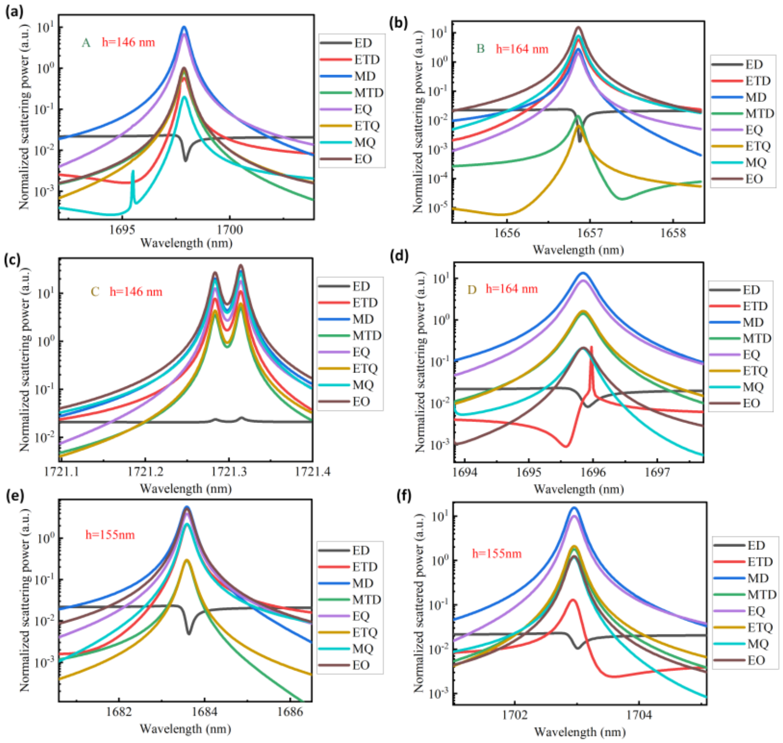

Fig. S4. Multipole decomposition of the scattering power at four points (A–D) in Fig. 9(b). (a)–(d) Correspond to Points A–D, respectively. (e) (f) is a point between A-B and C-D, respectively.

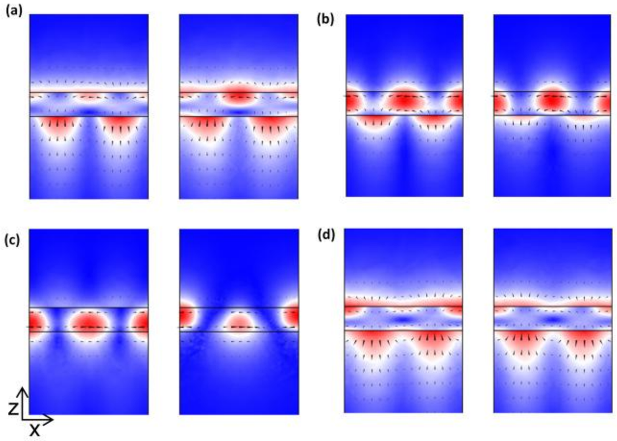

Fig. S5. Electric field distributions of the eigenmodes at the x-z cross-sections for points A–D in Fig. 9(b), where the arrows indicate the direction of the electric field. (a)–(d) Correspond to points A–D, respectively.

In this section Fig. S4 presents the multipole decomposition results of the scattered field at four points (A–D) in Fig. 9. As we move from Point A to Point B, the electromagnetic asymmetry κ increases. It can be observed from Fig. S4(a) that the contributions of the ETD, MQ and EO terms also increase, which is consistent with our previous inference. At Point D, the contributions of these two terms are even smaller; meanwhile, Point D exhibits a smaller κ value than Points A and B, which further verifies the validity of our inference.

For Point C, although the ETD and EO terms make relatively large contributions, the amplitudes of the scattered field terms from all multipoles are comparable. This leads to destructive interference among the multipoles, resulting in the formation of a resonant mode with a high quality factor at Point C (see Fig. 9(e)). Fig. S5 shows the eigenfield distributions of the vertical cross-sections at Points A–D. It can be seen that the electric field of the structure at Point C exhibits a relatively symmetric horizontal distribution (Fig. S5(c)), whereas the field distribution of the structure at Point B shifts toward the substrate to a significantly greater extent than that at Points A and C.

S6 Comparison between different works

In this section, we compare different works on nonlinearity-induced nonreciprocal transmission based on Silicon metasurfaces across the following aspects (Tab. S1): fabrication difficulty of the main surface structure, polarization sensitivity, incident angle of the excitation light, nonreciprocal intensity range (NRIR), and isolation at best insertion loss.

Tab. S1. Comparison between different works

**Structures**	**Fabrication difficulty**	**polarization sensitivity**	**Incident angle**	**$\chi^{(3)}$ (n_2)**	**NRIR**	**Isolation**
Periodically patterned Si grating in air [7]	Multi-steps etching of high alignment accuracy	Sensitive	Normal	2.79×10^{-18} m^2/V^2	4.77 dB (sim.)	-
Double layered Si nanosphere metasurface on glass [8]	Additional material transfer step	Insensitive	Normal	2.8×10^{-18} m^2/V^2	1.52 dB to 2.79 dB (sim.)	-
Grooved Si nanopillar tetramer metasurface in air [9]	Two etching steps	Insensitive	Normal	2.8×10^{-18} m^2/V^2	1.45 to 2.2 (1.61 dB to 3.42 dB) (sim.)	-
Amorphous Si grating on glass [10]	Single-step etching	Sensitive	Normal	10^{-14} m^2/V^2 (10^{-10} cm^2/W)	1.45 to 6.7 (2.07 dB to 8.26 dB) (exp.)	Max 10.7 dB (exp.)
Effective zero-index Si metasurface in air [11]	Single-step etching	Sensitive	Oblique	2.8×10^{-18} m^2/V^2	1.4 dB to 7.1 dB (sim.)	-
Grooved Si nanopillar metasurface in air [12]	Two etching steps	-	Normal	(1.12×10^{-12} cm^2/W)	1.63 to 3.56 (2.1 dB to 5.5 dB) (sim.)	15 dB to 34 dB (sim.)
Si plate-hole tetramer meta-surface on Silica [This work]	Single-step etching	Insensitive	Normal	2.8×10^{-18} m^2/V^2	1.65 to over 3.59 (1.17 dB to over 5.55 dB) (sim.)	8 dB to over 15 dB (sim.)

References

[1] E. A. Gurvitz, K. S. Ladutenko, P. A. Dergachev, A. B. Evlyukhin, A. E. Miroshnichenko and A. S. Shalin, "The High-Order Toroidal Moments and Anapole States in All-Dielectric Photonics", *Laser Photonics Rev.,* vol. 13, no. 5, pp. 2019. https://doi.org/10.1002/lpor.201800266.
[2] A. B. Evlyukhin, C. Reinhardt, E. Evlyukhin and B. N. Chichkov, "Multipole analysis of light scattering by arbitrary-shaped nanoparticles on a plane surface", *J. Opt. Soc. Am. B,* vol. 30, no. 10, pp. 2589-2598, 2013. https://doi.org/10.1364/JOSAB.30.002589.
[3] A. B. Evlyukhin, T. Fischer, C. Reinhardt and B. N. Chichkov, "Optical theorem and multipole scattering of light by arbitrarily shaped nanoparticles", *Phys. Rev. B,* vol. 94, no. 20, pp. 205434, 2016. https://doi.org/10.1103/PhysRevB.94.205434.
[4] K. Koshelev, S. Lepeshov, M. Liu, A. Bogdanov and Y. Kivshar, "Asymmetric Metasurfaces with High-Q Resonances Governed by Bound States in the Continuum", *Phys. Rev. Lett.,* vol. 121, no. 19, pp. 193903, 2018. https://doi.org/10.1103/PhysRevLett.121.193903.
[5] A. E. Miroshnichenko, S. Flach and Y. S. Kivshar, "Fano resonances in nanoscale structures", *Rev. Mod. Phys.,* vol. 82, no. 3, pp. 2257-2298, 2010. https://doi.org/10.1103/RevModPhys.82.2257.
[6] M. F. Limonov, M. V. Rybin, A. N. Poddubny and Y. S. Kivshar, "Fano resonances in photonics", *Nat. Photonics,* vol. 11, no. 9, pp. 543-554, 2017. https://doi.org/10.1038/nphoton.2017.142.
[7] M. Lawrence, D. R. Barton and J. A. Dionne, "Nonreciprocal Flat Optics with Silicon Metasurfaces", *Nano Lett.,* vol. 18, no. 2, pp. 1104-1109, 2018. https://doi.org/10.1021/acs.nanolett.7b04646.
[8] B. Jin and C. Argyropoulos, "Self-Induced Passive Nonreciprocal Transmission by Nonlinear Bifacial Dielectric Metasurfaces", *Phys. Rev. Appl.,* vol. 13, no. 5, pp. 054056, 2020. https://doi.org/10.1103/PhysRevApplied.13.054056.
[9] S. Chen, Y. Zeng, Z. Li, Y. Mao, X. Dai and Y. Xiang, "Passive nonreciprocal transmission and optical bistability based on polarization-independent bound states in the continuum", *Nanophotonics,* vol. 12, no. 18, pp. 3613-3621, 2023. https://doi.org/10.1515/nanoph-2023-0319.
[10] M. Cotrufo, A. Cordaro, D. L. Sounas, A. Polman and A. Alù, "Passive bias-free non-reciprocal metasurfaces based on thermally nonlinear quasi-bound states in the continuum", *Nat. Photonics,* vol. 18, no. 1, pp. 2024. https://doi.org/10.1038/s41566-023-01333-7.
[11] D. Bi, et al., "Significant Non-Reciprocal Transmission Achieved by Combining Nonlinear Near-Zero Index Materials with Bound States in the Continuum", *Laser Photonics Rev.,* vol. no. pp. 2401900, 2025. https://doi.org/https://doi.org/10.1002/lpor.202401900.
[12] Y. Liu, J. Li, R. Xiong and J. Hu, "Terahertz non-reciprocal transmission in silicon photonic crystal metasurfaces with high-Q quasi-bound states", *Opt. Express,* vol. 33, no. 4, pp. 8961-8970, 2025. https://doi.org/10.1364/OE.557967.
